# Arterial stiffness is not acutely modified by consumption of a caffeinated soft drink sweetened with high‐fructose corn syrup in young healthy adults

**DOI:** 10.14814/phy2.14777

**Published:** 2021-04-27

**Authors:** Jessica A. Freemas, Joel T. Greenshields, Tyler Baker, Stephen J. Carter, Blair D. Johnson, Zachary J. Schlader

**Affiliations:** ^1^ H.H. Morris Human Performance Laboratories Department of Kinesiology School of Public Health Indiana University Bloomington IN USA; ^2^ Cancer Prevention and Control Program Indiana University Melvin and Bren Simon Comprehensive Cancer Center Indianapolis IN USA

**Keywords:** blood pressure, hemodynamics, high‐fructose corn syrup, pulse wave velocity, soft drink

## Abstract

We tested the hypothesis that ingestion of a caffeinated soft drink sweetened with high‐fructose corn syrup acutely increases arterial stiffness. In a randomized counterbalanced, crossover design, fourteen healthy adults (25 ± 3 years, 6 women) reported to the laboratory for two experimental visits where 500 ml of tap water (H_2_O) or 500 ml of Mountain Dew® (a caffeinated soft drink sweetened with high‐fructose corn syrup (HFCS)) were consumed. Arterial stiffness (carotid‐to‐femoral pulse wave velocity (cfPWV)), peripheral and central blood pressures were measured pre‐consumption, 30 min post‐consumption, and 120 min post‐consumption. Prior to each measurement period, beat‐to‐beat hemodynamic measures were collected. Changes in heart rate, blood pressure, and cardiac output from pre‐consumption did not differ between trials at any timepoint (*p* ≥ 0.06). Moreover, changes in peripheral or central blood pressures from pre‐consumption did not differ between trials (*p* ≥ 0.84). Likewise, changes in cfPWV from pre‐consumption to 30 min post‐consumption (HFCS: 0.2 ± 0.3 m/s, H_2_O: 0.0 ± 0.3 m/s, *p* = 0.34) and 120 min post‐consumption (HFCS: 0.3 ± 0.4 m/s, H_2_O: 0.2 ± 0.3 m/s, *p* = 0.77) did not differ. Changes in aortic augmentation pressure, augmentation index, augmentation index corrected to a heart rate of 75 bpm, and reflection magnitude did not differ between conditions at 30 min post‐ (*p* ≥ 0.55) or 120 min post‐ (*p* ≥ 0.18) consumption. In healthy young adults, ingesting 500 ml of a commercially available caffeinated soft drink sweetened with high‐fructose corn syrup does not acutely change indices of arterial stiffness and wave reflection.

## Graphical abstract

This study investigated whether the ingestion of a caffeinated soft drink sweetened with high‐fructose corn syrup acutely modified arterial stiffness. In a randomized counterbalanced, crossover design, fourteen young healthy adults consumed 500 ml of tap water or a caffeinated soft drink sweetened with high‐fructose corn syrup, after which carotid‐to‐femoral pulse wave velocity was measured. This study found that ingesting 500 ml of a commercially available caffeinated soft drink sweetened with high‐fructose corn syrup does not acutely change indices of arterial stiffness.

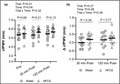

## 1 INTRODUCTION

Excessive intake of fructose is associated with a greater risk of developing cardiovascular disease (Malik & Hu, 2015). A major source of dietary fructose comes from ingestion of high‐fructose corn syrup in sugar‐sweetened beverages (Malik & Hu, 2015). Previously, our laboratory demonstrated that ingesting 500 ml of a commercially available caffeinated soft drink sweetened with high‐fructose corn syrup acutely elevates blood pressure during exercise in the heat (Chapman et al., 2019) and at rest (Chapman et al., 2020), which we subsequently isolated to the effects of high‐fructose corn syrup (Chapman et al., 2020). These findings are consistent with Brown et al. (Brown et al., 2008), who found that ingestion of a 100% fructose drink elevated blood pressure for up to 2 hr. Such changes were likely caused by increased cardiac output without a compensatory reduction in vascular resistance (Brown et al., 2008). Indeed, there is evidence revealing that dietary fructose shifts the vascular vasodilator‐vasoconstrictor balance toward vasoconstriction (Casey et al., 2008), which may prevent baroreflex mediated vasodilatory responses that would otherwise prevent a rise in blood pressure. Moreover, both the ingestion (Brown et al., 2008) and infusion (Schwarz et al., 1992) of fructose elevates heart rate, likely via activation of the sympathetic nervous system (Schwarz et al., 1992). Taken together, fructose metabolism appears to acutely alter cardiovascular hemodynamics, such that chronic exposure could elevate cardiovascular disease risk.

A compliant, healthy aorta dampens arterial pulsatility and protects the microvasculature from harmful hemodynamic patterns (Chirinos et al., 2019). High arterial stiffness is an independent risk factor for cardiovascular morbidity and mortality (Butlin & Qasem, 2017; Chirinos et al., 2019). Acute activation of the sympathetic nervous system (Casey et al., 2008) and elevations in blood pressure (Stewart et al., 2003), similar to those reported with fructose ingestion (Brown et al., 2008; Chapman et al., 2020), independently increase arterial stiffness. Moreover, the consumption of caffeine, which is often included in sugar‐sweetened beverages (Chou & Bell, 2007), acutely increases arterial stiffness (Harber et al., 2021; Le et al., 2012). Thus, it is possible that consuming a caffeinated beverage sweetened with high‐fructose corn syrup will acutely increase arterial stiffness. However, to our knowledge, this possibility has not been formally explored. Therefore, the purpose of this study was to test the hypothesis that ingesting a commercially available caffeinated soft drink sweetened with high‐fructose corn syrup (i.e., Mountain Dew) will acutely increase carotid‐to‐femoral pulse wave velocity (cfPWV)—the gold standard measure of arterial stiffness (Laurent et al., 2006).

## 2 METHODS

### 2.1 Participants

An *a priori* power analysis was performed with G‐Power version 3.1.9.4 (Faul et al., 2007) using data from our laboratory demonstrating that Mountain Dew sweetened with high‐fructose corn syrup increased renal vascular resistance by 0.6 ± 0.7 mmHg/cm/s (Chapman et al., 2020) (Cohen's *d_z_* = 0.86). Using this effect size, we estimated that thirteen participants were needed to detect differences in cfPWV using standard parameters of 1‐β = 0.80 and α = 0.05. Therefore, we recruited fourteen adults (25 ± 3 years, 6 women) to participate in this study. All participants self‐reported to be healthy, were non‐obese, normotensive, non‐tobacco users, reported no history of cardiovascular, kidney, autonomic, or metabolic diseases and were not taking any medications, except for hormonal contraceptives (n = 2). Participant physical characteristics were—height: 173 ± 9 cm, weight: 73.5 ± 13.9 kg, body mass index: 24.6 ± 3.6 m^2^/kg. Participants self‐reported to consume sugar‐sweetened soft drinks 1 ± 2 days/week. It is notable, however, that neither the inclusion nor exclusion criteria for this study considered habitual sugar‐sweetened soft drink consumption. All study procedures and informed consent documents were approved by the Indiana University Institutional Review Board. The study conformed to the standards set by the Declaration of Helsinki, except for registration in a database. Before completing any study‐related activities, each participant was fully informed of the experimental procedures and possible risks before providing informed, written consent.

### 2.2 Measurements

Height was measured with a stadiometer (Holtain Limited, Seritex, Wales, UK), and body weight was measured using a digital scale (Sauter, Balingen, Germany). Urine specific gravity was measured using refractometry (Atago, Tokyo, Japan). Heart rate was measured via a 3‐lead electrocardiogram (Datex‐Ohmeda, Instrumentarium Corp, Helsinki, Finland). Beat‐to‐beat blood pressure was measured via the Penaz method (Human NIBP Nano System, ADI Instruments, Colorado Springs, USA). Beat‐to‐beat blood pressure data were corrected to blood pressure measured via a manual auscultation of the brachial artery on their right arm. All manual blood pressure measurements were performed in duplicate by the same operator for both experimental trials within each participant. Pulse pressure was calculated by subtracting diastolic pressure from systolic pressure. Stroke volume was estimated from the arterial pressure waveform using the three element Windkessel model (Wesseling et al., 1993). Cardiac output was calculated as the product of stroke volume and heart rate, and total peripheral resistance was calculated as the quotient of mean arterial pressure and cardiac output.

Applanation tonometry (SphygmoCor XCEL, AtCor Medical, Sydney, Australia) was used to measure cfPWV by sequentially recording electrocardiogram gated carotid and femoral artery pressure waveforms (Butlin & Qasem, 2017). Transit distance between the carotid and femoral artery measurement locations was calculated as the surface distance from the suprasternal notch to the carotid and femoral recording sites measured with a flexible measuring tape. Carotid and femoral recording sites were marked indelible ink on the first assessment to ensure measurements were made from the same location each time. As a secondary analysis, a central aortic pressure waveform was constructed from pulse waves obtained at the radial artery using tonometry and a validated generalized transfer function (Pauca et al., 2001). The radial artery waveform was calibrated from brachial artery measurements of systolic and diastolic pressure, giving measure of aortic systolic, diastolic and mean pressures. Aortic pulse pressure was calculated as the difference between the diastolic and systolic aortic pressures. Indices of pulse wave reflection were also calculated from the synthesized aortic pressure waveforms. These measures are often interpreted as indicators of the overall systemic arterial stiffness and changes in vascular resistance downstream of the aorta (Butlin & Qasem, 2017). Aortic augmentation pressure was calculated as the difference between the second shoulder systolic peak and the first shoulder systolic peak. Aortic augmentation pressure was also expressed as a percentage of the aortic pulse pressure (termed the augmentation index). Heart rate was simultaneously determined from the aortic waveform and was used to standardize the augmentation index for a heart rate of 75 bpm. This was deemed necessary because heart rate is known to independently affect the augmentation index (Wilkinson et al., 2000). Finally, reflection magnitude was calculated as the ratio of the amplitude of the reflected (backward) wave and the amplitude of the forward traveling wave and is expressed as a percentage. Like completed for the measurement of cfPWV, the pulse wave analysis applanation tonometry measurement locations were marked with indelible ink to ensure that the measurement locations were the same at all measurement periods. All applanation tonometry measurements were performed by the same operator for both experimental trials within each participant and are presented as the average of two consistent high‐quality measurements at each time point. Measurement quality was determined based on the standards placed forth by the SphygmoCor software. For cfPWV, the two measurements did not differ by more than 0.5 m/s, which is consistent with the expert consensus for measuring pulse wave velocity (Van Bortel et al., 2012). High fidelity tonometry measurements could not be obtained in one participant during the HFCS trial pre‐consumption. Thus, data are presented as n = 13 for those measurement time points.

### 2.3 Study protocol

The present study employed a randomized, counterbalanced crossover design. Participants visited the laboratory on three occasions. Visit 1 included a screening and familiarization visit, while visits 2 and 3 were the experimental trials. Participants consumed either 500 ml of tap water (H_2_O) or 500 ml of Mountain Dew® (caffeinated beverage sweetened with high‐fructose corn syrup, HFCS) in their first visit and consumed the other beverage on their next experimental visit. Randomization was conducted in blocks of two to ensure trial order was counterbalanced. Outcome assessors were not blinded to the experimental conditions.

Participants reported to a temperature‐controlled laboratory (H_2_O: 23 ± 1°C; HFCS: 23 ± 1°C) at the same time of day for each experimental trial to control for any potential diurnal effects. Participants were instructed to refrain from ingesting caffeine in any form (e.g., coffee, energy drink), soft drinks, exercise, and alcohol for 12 hr whereas food was restricted for 2 hr prior to the experimental visits. The standardization of caffeine consumption may have resulted in caffeine withdrawal, but it was expected that the extent of this withdrawal would have been consistent for each visit. Although not formally quantified, participants were encouraged to maintain their normal exercise routines and diet throughout the duration of the experimental period. Female participants were tested during the first 10 days following menstruation (5 ± 3 days) to standardize measurements to the follicular phase of the menstrual cycle, when sex‐hormone levels are relatively low (Priest et al., 2018). A small sample of urine was collected from all participants to determine pre‐trial urine specific gravity was ≤1.020, ensuring participants were well hydrated prior to participating (measured urine specific gravity ‐ H_2_O: 1.012 ± 0.008, HFCS: 1.009 ± 0.005, *p* = 0.61) and to confirm a negative a pregnancy test for female participants. Participants then laid in the supine position for instrumentation.

**FIGURE 1:**
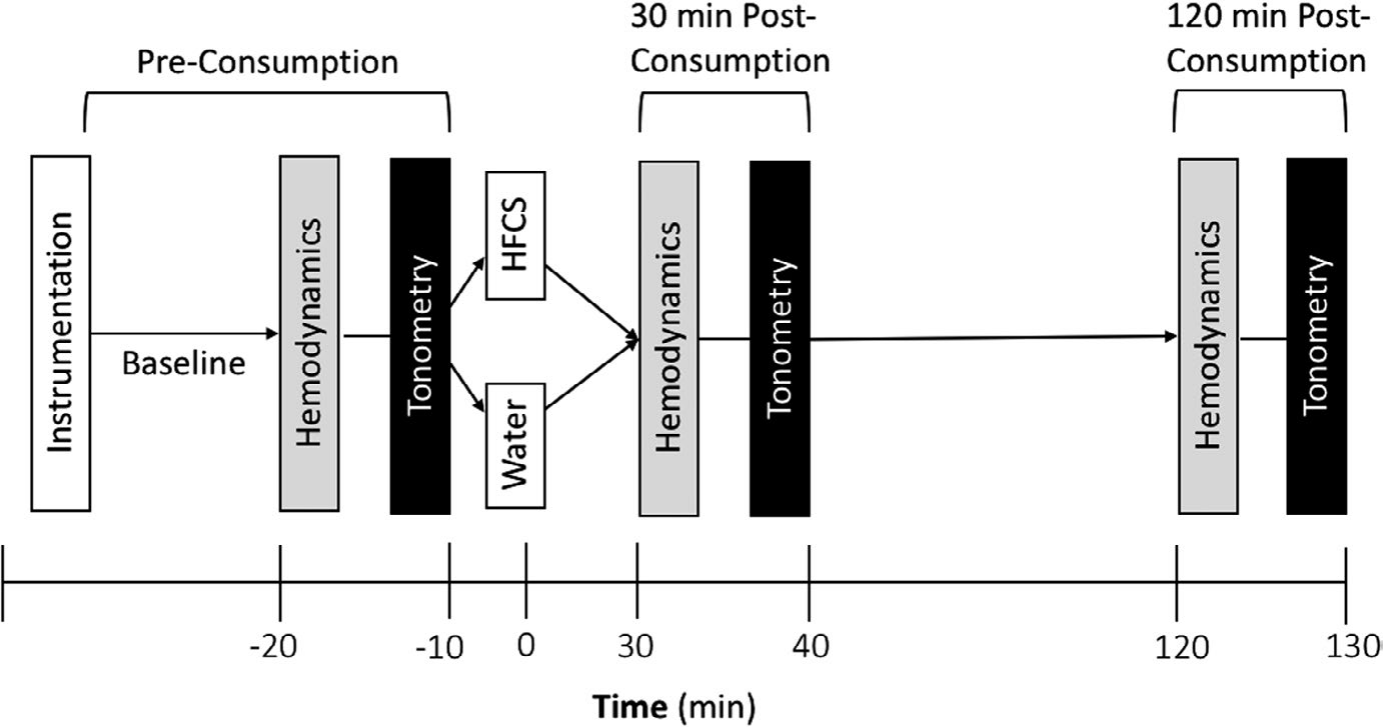
Diagram of study design. HFCS: high‐fructose corn syrup. Hemodynamic measures included cardiac output, stroke volume, total peripheral resistance, blood pressure, heart rate; Tonometry measures included the following: carotid‐to‐femoral pulse wave velocity, central blood pressure, central pulse pressure, heart rate, augmentation pressure, augmentation index, augmentation index at 75 bpm, reflection magnitude, forward wave magnitude, and reflected (backward) wave magnitude.

Following instrumentation, participants rested quietly in the supine position for 20 min after which pre‐consumption baseline steady‐state hemodynamic data were obtained over a 5 min period and then tonometry measurements were obtained (Figure 1). Next, participants assumed the semi‐recumbent position and were given 5 min to consume 500 ml of water (drinking duration: 1.7 ± 1.0 min) or Mountain Dew (drinking duration: 2.9 ± 1.1 min). Drinking duration was longer when drinking Mountain Dew compared to water (*p* < 0.01). Mountain Dew is a high‐calorie, high‐fructose, caffeinated soft drink sweetened with high‐fructose corn syrup. Mountain Dew was selected because it has one of the highest free fructose concentrations among commercially available soft drinks (Walker et al., 2014) and because our previous study isolated that some of the vascular effects of Mountain Dew were isolated to the high‐fructose corn syrup in the beverage (Chapman et al., 2020). In Mountain Dew, fructose and glucose each represent 59.5% and 39.7%, respectively, of the total sugar content (Walker et al., 2014). An analysis from Walker et al. (Walker et al., 2014), reported that ~36 g of fructose and ~25 g of glucose make up 500 ml of Mountain Dew. After drinking the water or Mountain Dew, participants then re‐assumed the supine position and steady‐state hemodynamics and tonometry measurements were obtained 30 min post‐consumption and again 120 min post‐consumption in each trial (Figure 1). These time points were chosen because ingestion of 500 ml of Mountain Dew elevates blood pressure 30 min following consumption during supine rest in a moderate thermal environment (Chapman et al., 2019) and increases in blood pressure have been observed to persist for ~120 min following consumption of a fructose beverage (Brown et al., 2008). Subjects were permitted to urinate, if needed, between the 30 and 120 min post‐consumption measurement time points.

### 2.4 Data and statistical analysis

Steady‐state hemodynamic data were collected at 1 kHz via a data acquisition system (LabChart, ADInstruments, Colorado Springs, CO). Steady‐state hemodynamics data were analyzed using the average over the last minute of each data collection period. Data were analyzed and presented as absolute values and as the change from pre‐consumption (Δ), the latter of which was deemed necessary to isolate the effect of beverage consumption on changes in the dependent variables over time. All data were statistically analyzed using Prism software (version 8, GraphPad Software, La Jolla, CA). A two‐way linear mixed model was used to examine any differences in the dependent variables over time (pre‐consumption, post‐consumption, and 120‐min post consumption) and between experimental trials (HFCS or H_2_O) with random effects to control for participant in the repeated measures design. From the results of the linear mixed models, estimated marginal means using Sidak's adjustment for multiple comparisons were used to test for differences between the HFCS and H_2_O trials at the measurement time points. *A priori* statistical significance was set at *p* ≤ 0.05. Data are reported as mean ± SD.

## 3 RESULTS

### 3.1 Steady‐state hemodynamics

Steady‐state hemodynamic data during the H_2_O and HFCS trials are presented in Table 1. There were no differences in cardiac output, stroke volume, total peripheral resistance, heart rate, or mean arterial pressure pre‐consumption (*p* ≥ 0.81). At 30 min post‐consumption, a difference in heart rate was observed between the HFCS and H_2_O trials (*p* = 0.05), but no other differences were shown in the remaining hemodynamic variables at 30 min post‐consumption (*p* > 0.13) or 120‐min post consumption (*p* ≥ 0.06).

**TABLE 1:** Steady state hemodynamic responses.

	H_2_O			HFCS			Linear mixed model output		
	Pre‐	30 min Post‐	120 min post‐	Pre‐	30 min Post‐	120 min Post‐	Trial	Time	Trial x Time
Heart rate (bpm)	56 ± 6	52 ± 6	59 ± 7	57 ± 8	56 ± 8^a^	60 ± 10	*p* = 0.10	*p* < 0.01	*p* = 0.22
Δ	‐	−4 ± 3	4 ± 7	‐	−1 ± 5	3 ± 8	*p* = 0.57	*p* < 0.01	*p* = 0.12
Stroke volume (ml)	80 ± 17	79 ± 14	86 ± 14	78 ± 16	83 ± 17	73 ± 14	*p* = 0.51	*p* = 0.65	*p* < 0.01
Δ	‐	−3 ± 11	6 ± 16	‐	6 ± 11	−2 ± 12	*p* = 0.91	*p* = 0.94	*p* = 0.01
Total peripheral resistance (mmHg/L/min)	21.3 ± 3.0	24.4 ± 5.5	20.3 ± 4.2	22.4 ± 7.1	22.3 ± 7.5	25.7 ± 8.6	*p* = 0.42	*p* = 0.57	*p* = 0.02
Δ	‐	3.2 ± 5.4	−1.0 ± 5.6	‐	−0.1 ± 4.6	2.5 ± 7.3	*p* = 0.91	*p* = 0.94	*p* = 0.01
Cardiac output (L/min)	4.4 ± 0.9	4.1 ± 0.7	5.0 ± 0.7	4.4 ± 1.2	4.7 ± 1.3	4.4 ± 1.1	*p* = 0.78	*p* = 0.09	*p* < 0.01
Δ	‐	−0.4 ± 0.7	0.6 ± 1.1	‐	0.3 ± 0.6	0.1 ± 1.0	*p* = 0.44	*p* < 0.01	*p* = 0.15
Mean arterial pressure (mmHg)	91 ± 8	95 ± 9	96 ± 6	93 ± 11	96 ± 9	101 ± 10	*p* = 0.14	*p* < 0.01	*p* = 0.63
Δ	‐	3 ± 9	6 ± 7	‐	4 ± 8	10 ± 10	*p* = 0.47	*p* = 0.06	*p* = 0.44

Mean ± SD, n = 14. Δ: absolute change from pre‐consumption. Data were analyzed using a two‐way linear mixed model.

^a^ Different from H_2_O based on Sidak's post hoc comparison (*p* = 0.05). All other post hoc comparisons *p* > 0.05. *p*‐values from the two‐way linear mixed model are reported.

### 3.2 Peripheral and central blood pressure

Peripheral and central blood pressure data are presented in Table 2. Peripheral systolic (*p* ≥ 0.99), diastolic (*p* ≥ 0.09), and mean arterial (*p* ≥ 0.39) pressures measured immediately preceding the calculation of central blood pressure were not different at pre‐consumption in the HFCS and H_2_O trials. This translated to no differences in central systolic (*p* ≥ 0.71) and mean arterial (*p* ≥ 0.22) pressures and a slightly elevated central diastolic (by 4 ± 5 mmHg, *p* = 0.05) pre‐consumption in the HFCS trial versus the H_2_O trial. There were no differences in peripheral (*p* ≥ 0.25) or central (*p* ≥ 0.13) blood pressure at 30 or 120 min post‐consumption between HFCS and H_2_O trials. Further, there were no differences in peripheral or central pulse pressures (*p* ≥ 0.99) at 30 or 120 min (*p* > 0.92) post‐consumption between HFCS and H_2_O trials.

**TABLE 2:** Peripheral and central blood pressure responses.

	H_2_O			HFCS			Linear mixed model output		
	Pre‐	30 min Post‐	120 min post‐	Pre‐	30 min Post‐	120 min Post‐	Trial	Time	Trial × Time
Peripheral DBP (mmHg)	69 ± 6	72 ± 7	74 ± 6	72 ± 7	74 ± 5	77 ± 6	*p* = 0.02	*p* = 0.01	*p* = 0.72
Δ	‐	2 ± 4	6 ± 5	‐	2 ± 5	4 ± 8	*p* = 0.55	*p* < 0.01	*p* = 0.51
Peripheral SBP (mmHg)	121 ± 8	126 ± 11	127 ± 10	122 ± 10	128 ± 10	128 ± 14	*p* = 0.32	*p* < 0.01	*p* = 0.83
Δ	‐	4 ± 8	6 ± 8	‐	7 ± 11	7 ± 13	*p* = 0.58	*p* = 0.65	*p* = 0.65
Peripheral MAP (mmHg)	86 ± 5	90 ± 7	92 ± 6	89 ± 6	92 ± 5	94 ± 8	*p* = 0.03	*p* < 0.01	*p* = 0.97
Δ	‐	3 ± 4	6 ± 5	‐	3 ± 6	5 ± 8	*p* > 0.99	*p* = 0.07	*p* = 0.54
Peripheral PP (mmHg)	52 ± 8	54 ± 9	52 ± 8	50 ± 9	54 ± 10	52 ± 12	*p* = 0.43	*p* = 0.13	*p* = 0.48
Δ	‐	2 ± 7	0 ± 6	‐	5 ± 9	3 ± 13	*p* = 0.35	*p* = 0.13	*p* = 0.91
Central DBP (mmHg)	70 ± 6	74 ± 8	75 ± 6	74 ± 6	75 ± 5	77 ± 6	*p* = 0.06	*p* = 0.06	*p* = 0.21
Δ	‐	3 ± 5	6 ± 5	‐	1 ± 5	3 ± 7	*p* = 0.12	*p* = 0.19	*p* = 0.99
Central SBP (mmHg)	105 ± 7	109 ± 9	109 ± 7	108 ± 7	110 ± 7	112 ± 11	*p* = 0.09	*p* = 0.06	*p* = 0.96
Δ	‐	2 ± 5	4 ± 6	‐	3 ± 7	4 ± 11	*p* = 0.92	*p* = 0.28	*p* = 0.70
Central MAP (mmHg)	82 ± 6	86 ± 7	86 ± 6	85 ± 6	87 ± 5	89 ± 7	*p* = 0.03	*p* = 0.10	*p* = 0.48
Δ	‐	3 ± 4	5 ± 5	‐	2 ± 5	3 ± 8	*p* = 0.70	*p* = 0.34	*p* = 0.72
Central PP (mmHg)	36 ± 6	34 ± 9	34 ± 5	34 ± 5	36 ± 6	34 ± 8	*p* = 0.55	*p* = 0.92	*p* = 0.34
Δ	‐	−1 ± 7	−2 ± 5	‐	2 ± 7	1 ± 8	*p* = 0.58	*p* = 0.93	*p* = 0.15

Mean ± SD, n = 14. Data were analyzed using a two‐way linear mixed model with Sidak post hoc comparisons (all *p* > 0.05). P‐values from the two‐way linear mixed model are reported.

MAP, mean arterial pressure; DBP, diastolic blood pressure; SBP, systolic blood pressure; PP, pulse pressure; Δ, absolute change from pre‐consumption.

### 3.3 Measures of arterial stiffness and wave reflection

**FIGURE 2:**
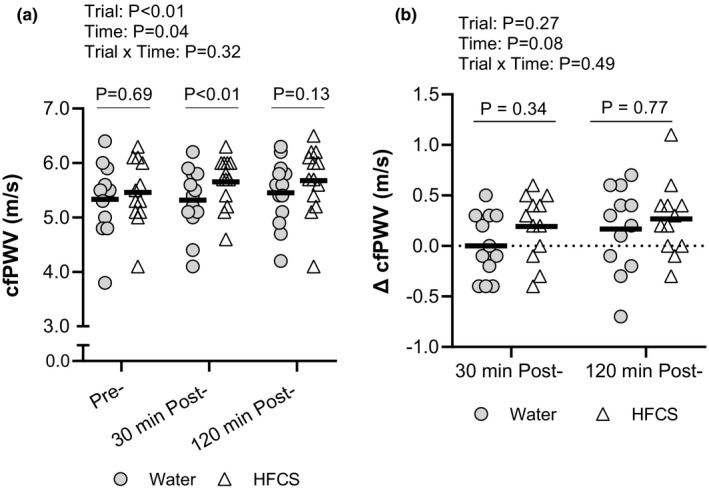
(a) Individual cfPWV measures between HFCS and H20 trials at pre‐consumption (n = 13), 30 min post‐consumption (n = 14), and 120‐min post‐consumption (n = 14). (b) The change from pre‐consumption to 30 min post‐ and to 120 min post‐consumption in cfPWV measures (n = 13). Thick lines indicate the mean. Data were analyzed using a two‐way linear mixed model with Sidak post hoc comparisons. *P*‐values from the two‐way linear mixed model and post hoc comparisons are reported.

**FIGURE 3:**
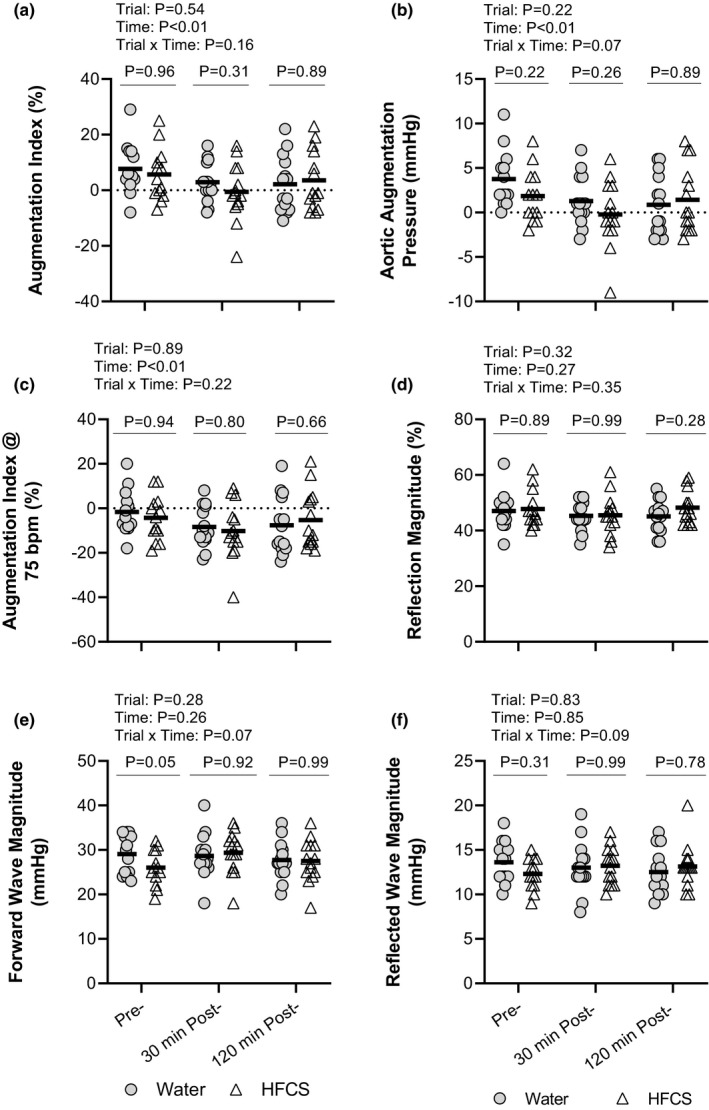
(a) Individual measures of augmentation index between HFCS and H20 trials at pre‐consumption (n = 13), 30 min post‐consumption (n = 14), and 120‐min post‐consumption (n = 14). (b) Individual measures of augmentation pressure between HFCS and H20 trials at pre‐consumption (n = 13), 30 min post‐consumption (n = 14), and 120‐min post‐consumption (n = 14). (c) Individual measures of augmentation index at 75 bpm between HFCS and H20 trials at pre‐consumption (n = 13), 30 min post‐consumption (n = 14), and 120‐min post‐consumption (n = 14). (d) Individual measures of the reflection magnitude between HFCS and H20 trials at pre‐consumption (n = 13), 30 min post‐consumption (n = 14), and 120‐min post‐consumption (n = 14). (e) Individual measures of forward wave magnitude between HFCS and H20 trials at pre‐consumption (n = 13), 30 min post‐consumption (n = 14), and 120‐min post‐consumption (n = 14). (f) Individual measures of reflected (backward) wave magnitude between HFCS and H20 trials at pre‐consumption (n = 13), 30 min post‐consumption (n = 14), and 120‐min post‐consumption (n = 14). Thick lines indicate the mean. Data were analyzed using a two‐way linear mixed model with Sidak post hoc comparisons. *P*‐values from the two‐way linear mixed model and post hoc comparisons are reported.

There were no differences in cfPWV (*p* = 0.69, Figure 2a), augmentation index (*p* = 0.96, Figure 3a) aortic augmentation pressure (*p* = 0.22, Figure 3b), augmentation index at 75 bpm (*p* = 0.94, Figure 3c), reflection magnitude (*p* = 0.89, Figure 3d), or reflected wave magnitude (*p* = 0.31, Figure 3f) pre‐consumption between trials. However, forward wave magnitude was 4.8 ± 7.4 mmHg higher at pre‐consumption in the HFCS trial (*p* = 0.05). cfPWV at 30 min post‐consumption was higher in the HFCS trial (5.7 ± 0.6 m/s) compared with the H_2_O trial (5.3 ± 0.6 m/s, *p* = 0.01, Figure 2a), but cfPWV was not different between the HFCS and H_2_O at 120 min post‐consumption (HFCS: 5.7 ± 0.6 m/s, H_2_O: 5.5 ± 0.6 m/s, *p* = 0.13, Figure 2a). Changes in cfPWV from pre‐consumption did not differ between the HFCS and H_2_O trials at 30 min post‐ (HFCS: 0.2 ± 0.3 m/s, H_2_O 0.0 ± 0.3 m/s, *p* = 0.34 Figure 2b) and 120 min post‐consumption (HFCS: 0.3 ± 0.4 m/s, H_2_O: 0.2 ± 0.3 m/s, *p* = 0.77, Figure 2b). Aortic augmentation pressure, augmentation index, augmentation index corrected to a heart rate of 75 bpm, reflection magnitude, forward wave magnitude, and reflected wave magnitude did not differ between the HFCS and H_2_O trials at 30 min post‐ (*p* ≥ 0.26) or 120 min post‐consumption (*p* ≥ 0.28, Figure 3). The change from pre‐consumption to 30 min post‐consumption did not differ between the HFCS and H_2_O trials for the augmentation index (HFCS: −6.9 ± 7.3%, H_2_O: −4.5 ± 7.5%, *p* = 0.55) aortic augmentation pressure (HFCS: −2.3 ± 2.7 mmHg, H_2_O: −2.4 ± 3.7 mmHg, *p* = 0.99), augmentation index at 75 bpm (HFCS: −7.2 ± 7.7%, H_2_O: −6.3 ± 7.6%, *p* = 0.89), reflection magnitude (HFCS: −3.1 ± 5.0%, H_2_O: −1.5 ± 8.2%, *p* = 0.70), forward wave magnitude (HFCS: +3.7 ± 5.3 mmHg, H_2_O: −0.4 ± 5.5 mmHg, *p* = 0.10), or reflected wave magnitude (HFCS: +0.8 ± 2.1 mmHg, H_2_O: −0.5 ± 2.5 mmHg, *p* = 0.33). Likewise, the change from pre‐consumption to 120 min post‐consumption did not differ between the HFCS and H_2_O trials for the augmentation index (HFCS: −3.6 ± 5.0%, H_2_O: −5.2 ± 10.6%, *p* = 0.69) aortic augmentation pressure (HFCS: −0.9 ± 2.0 mmHg, H_2_O: −2.8 ± 4.6 mmHg, *p* = 0.18), augmentation index at 75 bpm (HFCS: −3.0 ± 5.6%, H_2_O: −5.4 ± 12.6%, *p* = 0.43), reflection magnitude (HFCS: −0.2 ± 3.5%, H_2_O: −1.5 ± 8.8%, *p* = 0.68), forward wave magnitude (HFCS: +1.7 ± 6.4 mmHg, H_2_O: −1.5 ± 4.0 mmHg, *p* = 0.21), or reflected wave magnitude (HFCS: +0.8 ± 2.8 mmHg, H_2_O: −1.1 ± 2.8 mmHg, *p* = 0.10).

## 4 DISCUSSION

The present study tested the hypothesis that consumption of 500 ml of a commercially available caffeinated soft drink sweetened with high‐fructose corn syrup acutely increases arterial stiffness. Indeed, we observed that absolute cfPWV was higher in the HFCS trial compared with the H_2_O trial 30 min after beverage consumption (Figure 2a). That said, the change in cfPWV from pre‐consumption did not differ between the HFCS and H_2_O trials at either 30 or 120 min post‐consumption (Figure 2b). Moreover, indices of wave reflection, such as augmentation index, augmentation pressure, augmentation index at 75 bpm, reflection magnitude, forward wave magnitude, and reflected wave magnitude (Figure 3), did not differ following beverage consumption in the HFCS and H_2_O trials, which remained true whether analyzed as absolute values or the change from pre‐consumption. Amalgamation of these findings suggests that the difference in cfPWV between the HFCS and H_2_O trials at 30 min post beverage consumption time point is likely spurious and contributed to by subtle, albeit not statistically significant, differences in cfPWV pre‐consumption. Therefore, our data do not support our hypothesis. It is also notable that we did not observe any meaningful differences in the measured hemodynamic parameters, such as cardiac output (Table 1), total peripheral resistance (Table 1), or peripheral or central blood pressure (Table 2). Collectively, our findings indicate that, compared with drinking an equivalent volume of water, consumption of 500 ml of a commercially available caffeinated soft drink sweetened with high‐fructose corn syrup has little acute effect on arterial stiffness and systemic hemodynamics under the conditions employed herein.

Previous studies demonstrate an association between diets high in fructose and increases in cardiovascular morbidity and mortality (Chapman et al., 2019; Institute of Medicine (US) Committee on Military Nutrition Research, 2021; Priest et al., 2018). High arterial stiffness is an independent risk factor for cardiovascular morbidity and mortality (Butlin & Qasem, 2017; Chirinos et al., 2019). Determinants of arterial stiffness are both anatomical and physiological in nature. Anatomical factors include structural elements of the arteries (e.g., collagen content), which are unlikely to be modified in an acute setting, such as that employed in the present study. Thus, there are likely three physiological reasons why we did not observe any changes in arterial stiffness or wave reflection in the HFCS compared with the H_2_O trial (Figures 2 and 3).

First, acute elevations in blood pressure increase arterial stiffness (Stewart et al., 2003). Previous evidence indicates that fructose ingestion (Brown et al., 2008), caffeine intake (Mahmud & Feely, 2001) and consumption of a caffeinated soft drink sweetened with high‐fructose corn syrup (Chapman et al., 2020) acutely increase blood pressure in healthy young adults, some of whom have reported consuming 1–2 soft drinks per week (Chapman et al., 2020), which is similar to the participants in the present study. Thus, we expected to observe modest elevations in blood pressure in the HFCS trial. To the contrary, however, neither peripheral or central blood pressure differed at any time between the HFCS and H_2_O trials (Table 2). That said, our findings are consistent with previous observations that the infusion of fructose (Vollenweider et al., 1993) and consumption of a beverage sweetened with high‐fructose corn syrup (Le et al., 2012) did not modify mean arterial pressure. The reason for the blood pressure differences between our study and previous studies (Brown et al., 2008; Chapman et al., 2020) is largely unclear. A previous study demonstrated that intake of 60 g of fructose elevated blood pressure in young healthy adults that was sustained for 120 min (Brown et al., 2008). Given that this dose is almost double that given in the current study (~36 g (35) vs. 60 g) it may be that the dose of fructose was not sufficient to evoke a pressor response in the current study. Moreover, it is possible that the dose of caffeine was not sufficient to elevate blood pressure in young healthy adults, such that consistent rises in blood pressure are often observed with doses of caffeine exceeding 90 mg (Nurminen et al., 1999), while increases in arterial stiffness are observed with a dose of 250 mg or higher (Harber et al., 2021; Le et al., 2012). Notably, these doses exceed the ~77 mg of caffeine contained within 500 ml of Mountain Dew used in the current study (Chapman et al., 2020). Nevertheless, none of these previous studies help explain the differences in blood pressure between our previous study (Chapman et al., 2020) and current study, particularly considering that the beverage type, beverage volume, the timing of the measurements, experimental controls, and the measurement procedures were identical between the two studies. That said, the lack of pressor effect caused by drinking a caffeinated soft drink sweetened with high‐fructose corn syrup in the present study is likely a function of a negligible impact of the beverage on the acute activation of the sympathetic nervous system and/or circulating vasoactive compounds, both of which are discussed below.

Second, sympathetic nervous system activation is independently associated with arterial stiffening (Swierblewska et al., 2010). This is perhaps best demonstrated in the observation that the cold pressor test, a sympathoexcitatory stimulus, acutely increases cfPWV, although these changes in cfPWV occurred alongside increases in both peripheral and central blood pressure (Bock et al., 2019). Nevertheless, both the ingestion (Brown et al., 2008) and infusion (Schwarz et al., 1992) of fructose elevates heart rate, which likely occurs via activation of the sympathetic nervous system given that the rise in heart rate during fructose infusion is attenuated by propranolol, a β‐adrenoreceptor antagonist (Schwarz et al., 1992). That said, it is unclear whether this sympathetic activation is systemic or specific to cardiac tissues, particularly given evidence that muscle sympathetic nerve activity is not affected by fructose infusion (Vollenweider et al., 1993). In the present study, absolute heart rate as slightly higher in the HFCS trial 30 min post‐consumption, but this was likely due to reductions in heart rate in the H_2_O trial. Thus, we have no indirect evidence for differential sympathetic activation between the HFCS and H_2_O trials, given that neither changes in heart rate, stroke volume, nor total peripheral resistance differed between trials at any time (Table 1). The hemodynamic data presented herein are consistent with previous work from our laboratory employing the same experimental beverage, measurements, and measurement timeline (Chapman et al., 2020). Thus, it is unlikely that consumption of a caffeinated soft drink sweetened with high‐fructose corn syrup acutely activates the sympathetic nervous system.

Third, increases in circulating endogenous vasoconstrictor agents (e.g., angiotensin II) or decreases in vasodilators (e.g., nitric oxide) are known to acutely increase arterial stiffness (Kinlay et al., 2001). In the present study, we did not observe any hemodynamic changes consistent with alterations in the systemic vasodilator‐vasoconstrictor balance (e.g., increases in total peripheral resistance) (Table 1). Thus, it is perhaps not surprising that consumption of a caffeinated soft drink sweetened with high‐fructose corn syrup did not modify arterial stiffness or indices of wave reflection. Nevertheless, it remains possible that high fructose intake and/or caffeine could exert negative effects on specific vascular beds, that may not always manifest in acute changes in systemic hemodynamics. For instance, our laboratory has observed increases in renal vascular resistance 30 min after ingestion of a commercially available caffeinated soft drink sweetened with high‐fructose corn syrup that were accompanied by a minimal changes in systemic hemodynamics (Chapman et al., 2020). We subsequently speculated that the beverage may have shifted the vasodilator‐vasoconstrictor balance in the kidneys toward vasoconstriction by stimulating vasoconstrictor substance release, such as vasopressin and uric acid (Chapman et al., 2020). In support of this conjecture, Glushakova et al. reported that ingestion of fructose decreased nitric oxide derived from endothelial nitric oxide synthase (Glushakova et al., 2008), which based on the findings presented herein is likely specific to the kidneys. Furthermore, Kamide et al. demonstrated that fructose upregulates angiotensin II receptors that can promote vasoconstriction (Kamide et al., 2002) and are particularly prevalent in the renal vasculature. Collectively, given that we observed no meaningful differences in any of our arterial stiffness or hemodynamic parameters in the HFCS versus the H_2_O trial, it is unlikely that any acute local shifts in the vasodilator‐vasoconstrictor balance are sufficient to modify systemic cardiovascular responses.

### 4.1 Experimental considerations

Our participants were young, healthy individuals who consumed approximately one sugar‐sweetened soft drink per week. Approximately half of American adults consumed one or more sugar‐sweetened beverages on any given day (Kit et al., 2013). Thus, our participants fall within the lower half of the soft drink consumption spectrum. Therefore, our participant population may have contributed to our findings. If we had recruited people who regularly consume soft drinks, perhaps we would have observed systemic hemodynamic changes in the HFCS versus the H_2_O trial. This is supported by evidence that chronic consumption of sugar‐sweetened beverages is associated with a greater risk of developing cardiovascular morbidity and mortality (Arsenault et al., 2017; Mahmud & Feely, 2001; Pauca et al., 2001). Moreover, it is possible that recruiting people who had never consumed soft drinks may have also resulted in differential hemodynamic responses in the HFCS compared with the H_2_O trial, perhaps due to the novelty of the high‐fructose corn syrup in the soft drink.

Our participants arrived at the laboratory following a 2 hr fast and after abstaining from caffeine for at least 12 hr. This is important given evidence that fructose consumption in the fed state may augment cardiovascular risk owing to the potentiation of the postprandial increase in plasma triglycerides that is dependent on hepatic glycogen stores (Hengist et al., 2019). Thus, it is possible that the acute cardiovascular response to consumption of a caffeinated soft drink sweetened with high‐fructose corn syrup is modified by hepatic glycogen status. However, this possibility remains to be explored. Moreover, it is also possible that the 2 hr duration of fasting before arrival at the laboratory was too short to ensure that prior food and/or beverage intake did not confound our findings. We think this possibility is unlikely given that the dependent variables at pre‐consumption did not differ between trials. However, we would be remiss not to mention that this may have attributed to the variability observed as this time point. Similarly, having subjects abstain from caffeine for 12 hr was chosen because the half‐life of caffeine in the plasma of healthy adults is ~5 hr (Institute of Medicine (US) Committee on Military Nutrition Research, 2021). However, this may have been too short to ensure that any effects of prior caffeine consumption did not confound our data.

To enhance the external validity of our study, we employed a commercially available caffeinated soft drink, as we previously reported (Chapman et al., 2019, 2020). The downside to this approach, however, is that we could not confidently state whether a particular constituent of the beverage contributed to the findings presented herein. Thus, it remains possible that had we used a beverage containing a higher concentration of fructose (i.e., >60%), we may have observed systemic hemodynamic changes, as has been observed previously (Brown et al., 2008).

In the current study, we did not collect blood samples to elucidate the effect of consuming a caffeinated beverage on circulating vasoactive compounds that may have modified systemic cardiovascular responses. We do not think that this is a limitation because even if circulating vasodilator/vasoconstrictors were modified by consuming 500 ml a caffeinated soft drink sweetened with high‐fructose corn syrup, these changes did not translate to changes in arterial stiffness or systemic hemodynamics.

### 4.2 Perspectives

The present study demonstrates that, compared with an equivalent volume of water, consumption of 500 ml of a commercially available caffeinated soft drink sweetened with high‐fructose corn syrup does not modify arterial stiffness or systemic hemodynamics over the subsequent 120 min of observation in young healthy adults. Thus, under the experimental conditions employed herein, consumption of a soft drink sweetened with high‐fructose corn syrup does not acutely modify factors associated with heighted cardiovascular risk. This conclusion is likely contributed to by the healthy and lean participant population that completed this study in a relatively fasted state. Moreover, it remains plausible chronic intake of beverages sweetened with high‐fructose corn syrup may lead to modification of cardiovascular responses. This possibility is highlighted by a recent study demonstrating that one week of daily consumption of a glucose‐sweetened beverage induced vascular endothelial dysfunction independent of changes in fasting glucose or insulin concentrations (Bock et al., 2020). Future studies should focus on repeated consumption of soft drinks sweetened with high‐fructose corn syrup and the subsequent effects on indices of arterial stiffness and systemic hemodynamics in both healthy and populations with an elevated cardiovascular risk profile, such as those with hypertension, diabetes, or obesity.

## 5 CONCLUSIONS

Compared with drinking an equivalent volume of water, consumption of 500 ml of a commercially available caffeinated soft drink sweetened with high‐fructose corn syrup did not acutely modify arterial stiffness or systemic hemodynamics in fasted, young healthy adults.

## DISCLOSURE

No conflicts of interest, financial or otherwise, are declared by the authors.

## AUTHOR CONTRIBUTIONS

SJC, BDJ, and ZJS contributed to conception and design; JAF, JTG, TB, ZJS performed experiments; JAF, JTG, and ZJS analyzed data; JAF, SJC, JTG, BDJ, and ZJS interpreted results; JAF prepared figures; JAF and ZJS drafted manuscript; All authors edited and revised manuscript; approved final version of manuscript.
